# 5-methylcytosine RNA modification regulators-based patterns and features of immune microenvironment in acute myeloid leukemia

**DOI:** 10.18632/aging.205484

**Published:** 2024-01-25

**Authors:** Yuhong Ding, Akhilesh K. Bajpai, Fengxia Wu, Weihua Lu, Lin Xu, Jiawei Mao, Qiang Li, Qi Pan, Lu Lu, Xinfeng Wang

**Affiliations:** 1 Department of Hematology, The Affiliated Hospital of Nantong University, Jiangsu 226000, China; 2 Department of Genetics, Genomics and Informatics University of Tennessee Health Science Cente, Memphis, TN 38163, USA; 3 Department of Hematology and Oncology, The Branch Affiliated Hospital of Nantong University, Jiangsu 226000, China

**Keywords:** acute myeloid leukemia, 5-methylcytosine, immune infiltration, prognostic model, qPCR

## Abstract

Acute myeloid leukemia (AML) is a highly heterogeneous malignant disease of the blood cell. The current therapies for AML are unsatisfactory and the molecular mechanisms underlying AML are unclear. 5-methylcytosine (m5C) is an important posttranscriptional modification of mRNA, and is involved in the regulation of mRNA stability, translation, and other aspects of RNA metabolism. However, based on our knowledge of published literature, the role of the m5C regulators has not been explored in AML till date. In this study, we clarified the expression and gene variants of m5C regulators in AML and found that most m5C regulators were differentially expressed and correlated with disease prognosis. We also found that the methylation status of certain m5C regulators (e.g., *DNMT3A*, *DNMT3B*) affects the survival of AML patients. Two m5C modification subtypes, and high- and low-risk subgroups identified based on the expression of m5C regulators showed significant differences in the prognosis as well as immune cell infiltration. In addition, most of the m5C regulators were found to be correlated with miRNA expression in AML, as well as IC50 values of many drugs. The miRNA and GSVA analysis were used to identify the different miRNAs and KEGG or hallmark pathways between high- and low-risk subgroups. We also built a prognostic model based on m5C regulators, which was validated by two GSE databases. To verify the reliability of our analysis and conclusions, qPCR was used to identify the expressions of m5C regulators between normal and AML. In summary, we comprehensively explored the molecular characteristics of m5C regulators and built a prognostic model in AML. We proposed new mechanistic insights into the role of m5C in multiple databases and clinical data, which may pave novel ways for the development of therapeutic strategies.

## INTRODUCTION

Acute myeloid leukemia (AML) is a fatal hematopoietic disease, which is characterized by excessive proliferation of hematopoietic progenitor cells during the differentiation and development of myeloid cells [1]. Clinical manifestations include infection, hemorrhage, anemia and infiltration of extramedullary tissues and organs, and the disease progresses rapidly with a natural course of only a few weeks to a few months [2]. The incidence rate increases with age and there are about 20,000 new cases of AML diagnosed each year in the USA [3]. AML places a significant financial burden on society and causes heavy emotional trauma to many families [4]. Despite advances in therapy (e.g., Venetoclax, Azacitidine, Gilteritinib, Glasdegib) [5–8], the AML patients remain have poor survival rates (5-year survival = 24%). AML pathogenesis is a complex multistep process: not only related to gene mutation, but also affected by epigenetic modifications (DNA methylation and histone modifications) [9] and posttranscriptional regulation (m6A methylation and alternative polyadenylation) [10–13]. Understanding the mechanisms of AML molecular pathogenesis can support the development of better therapeutic strategies.

5-methylcytosine (m5C) is a common RNA modification in eukaryotic cells [14, 15], which occurs in mRNAs, tRNAs, rRNAs and ncRNAs [16, 17]. The dynamic m5C regulation depends on three kinds of regulatory factors, including methyltransferases (“writers”), demethylases (“erasers”), and m5C binding proteins (“readers”). “Writer” includes 11 factors (*NOP2, NSUN2, NSUN3, NSUN4, NSUN5, NSUN6, NSUN7, DNMT1, DNMT3A, DNMT3B*, and *TRDMT1*), “Erasers” (*TET1*, *TET2*, *TET3*, and *ALKBH1*) change the status of m5C modification and “Readers” (*ALYREF* and *YBX1*) recognize and bind to the m5C modification sites [17–20]. The m5C modification plays a critical regulatory role in multiple aspects of biological processes, including RNA export, RNA stability, and translation [21]. Recent studies have shown that m5C modification plays an important role in the development and progression of a variety of tumors [22, 23]. However, the expression pattern and molecular mechanisms of m5C regulators remain unclear in AML.

In this study, we retrospectively analyzed 17 m5C regulators in AML based on their expression profiles, mutation annotation data and copy number alteration data collected from The Cancer Genome Atlas (TCGA) database. We found that these m5C regulators were differentially expressed in AML, and mutations and copy number variations (CNVs) of some of the m5C regulators were significantly associated with the survival of AML patients. Furthermore, based on these 17 m5C regulators, AML patients were classified into two clusters. Subsequently, we identified a 6-gene signature and constructed a m5C-regulators risk model for assessing the risk of AML patients. We also analyzed the differences in immune cell infiltration in high- and low-risk groups and explored the immunological mechanisms underlying differential prognosis of these groups. Finally, we analyzed the relationship of m5C regulators with miRNAs and drug sensitivity to provide a reference for exploring AML mechanisms and develop possible novel epigenetic therapies.

## RESULTS

### Identification of expression levels and clinical correlation of m5C regulators in acute myeloid leukemia (AML)

**Figure 1:**
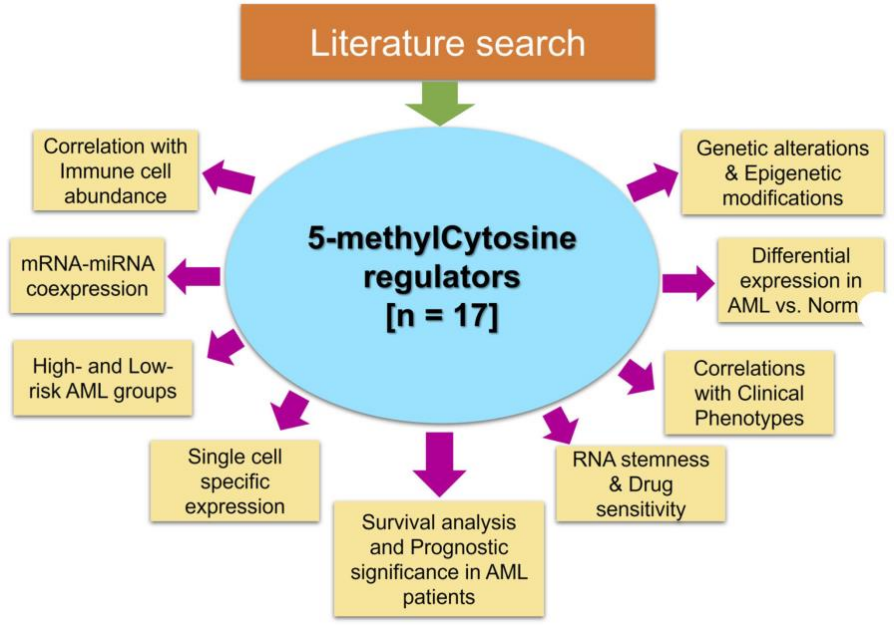
Overall flow of the analysis.

**Figure 2:**
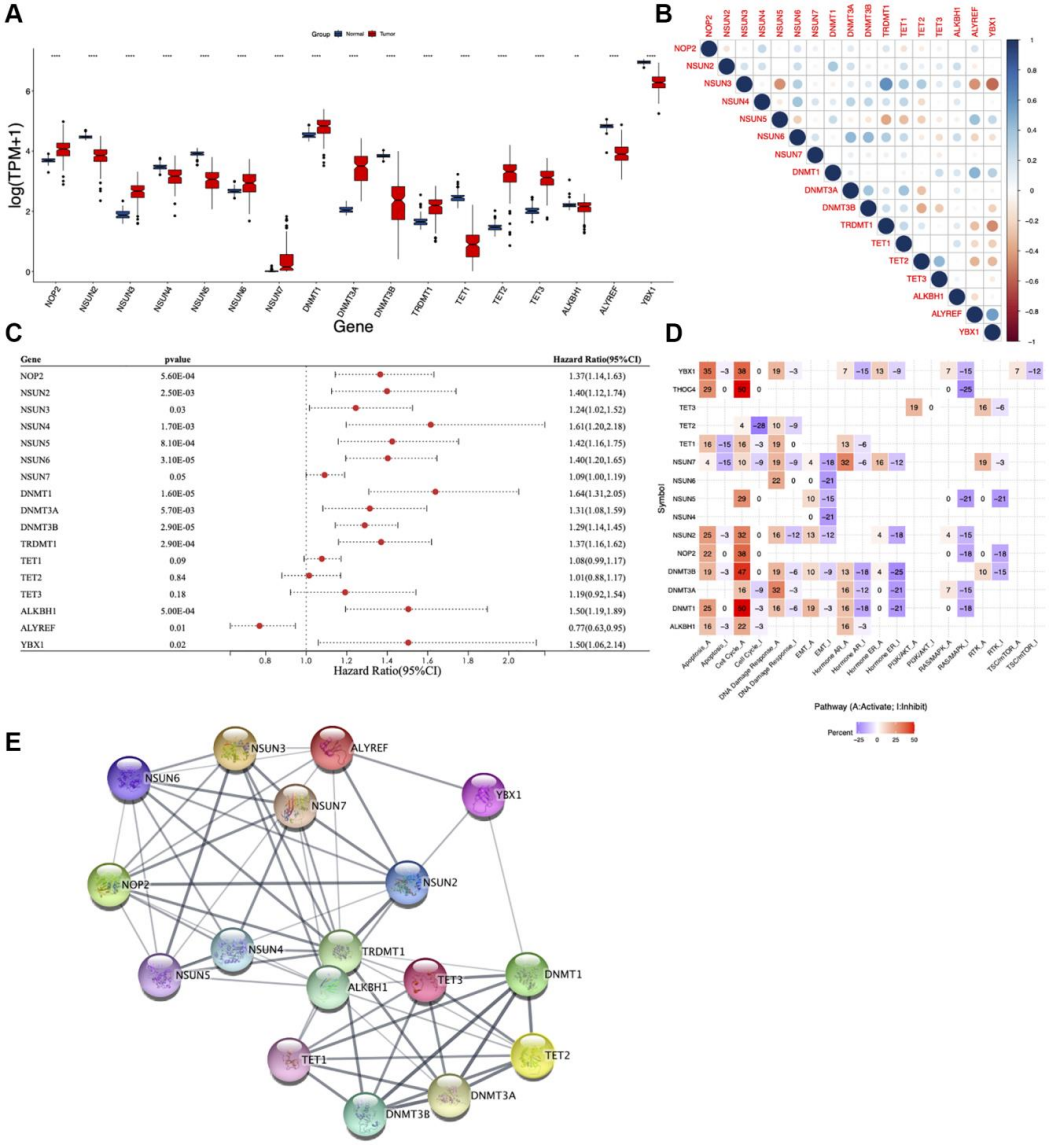
**Expression, correlation, and function of m5C regulators.** (**A**) Boxplot showing differential expression of m5C regulators in AML and normal samples. (**B**) Pearson correlation analysis, (**C**) univariate Cox regression analysis, (**D**) pathway activity analysis and (**E**) protein-protein interaction (PPI) analysis of m5C regulators. ^*^p < 0.05, ^**^p < 0.01, ^***^p < 0.001.

According to the existing literature reports [16, 17], seventeen m5C regulators (*NOP2, NSUN2, NSUN3, NSUN4, NSUN5, NSUN6, NSUN7, DNMT1, DNMT3A, DNMT3B, TRDMT1, TET1, TET2, TET3, ALKBH1, ALYREF, YBX1*) were identified and explored in this study through various approaches (Figure 1). We first used inSilicoMerging package to merge datasets from GTEx and TCGA databases followed by combat function in SVA package to remove the batch effects, and then compared the expression levels of these regulators between AML and normal samples. Interestingly, we found unexpectedly high expression of nine m5C regulators in the tumor group (*NOP2*, *NSUN3*, *NSUN6*, *NSUN7*, *DNMT1*, *DNMT3A*, *TRDMT1*, *TET2*, and *TET3*), indicating their essential role in AML pathogenesis (Figure 2A). Furthermore, correlation analysis revealed that most of the m5C regulators positively correlated with each other, while *NSUN5*, *ALYREF* and *YBX1* had negative correlations with other m5C regulators (Figure 2B). To clarify whether m5C regulators could affect the prognosis of AML patients, we performed univariate Cox regression analysis. Our results demonstrated that higher expression of m5C regulators (except for *TET1, TET2, TET3, ALYREF*) was associated with worse prognosis (Figure 2C). Further, the pathway activity analysis showed that most of the studied m5C regulators activated apoptosis, cell cycle and DNA damage response signaling pathways, whereas inhibited EMT, hormone ER and RAS/MAPK signaling pathways (Figure 2D). Protein-protein interaction (PPI) analysis showed that there are implicated relationships among these m5C regulators (Figure 2E).

**Figure 3:**
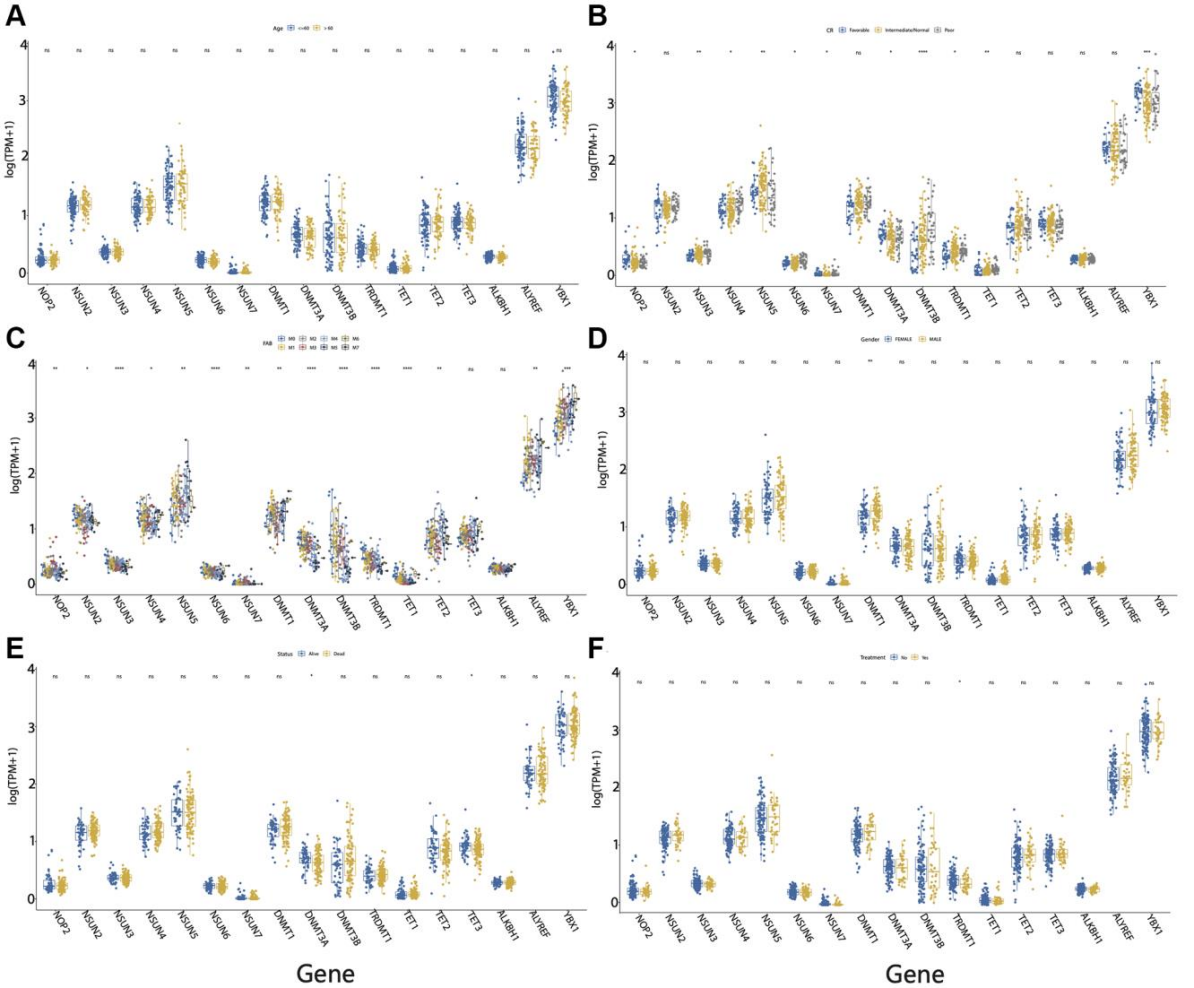
**Clinical characteristics of m5C regulators in acute myeloid leukemia (AML).** Expression of m5C regulators between (**A**) age groups, (**B**) CR groups, (**C**) FAB groups, (**D**) male and female, (**E**) survival status, and (**F**) before and after treatment in AML patients. ^*^p < 0.05, ^**^p < 0.01, ^***^p < 0.001.

It is unclear whether different clinical indicators can affect the expression of m5C regulators. Our results suggested that the expression of m5C regulators did not differ across the age groups (Figure 3A). In different CR groups, *NOP2, NSUN3, NSUN4, NSUN5, NSUN6, NSUN7, DNMT3A, DNMT3B, TRDMT1, TET1* and *YBX1* were found to be significantly different (Figure 3B). Furthermore, most m5C regulators (except for *TET3* and *ALKBH1*) were significantly differentially expressed in different FAB groups (Figure 3C). In terms of gender, we did not observe any significant difference in the expression of m5C regulators (except for *DNMT1*) between male and female patients. (Figure 3D). The expression of only *DNMT3A* and *TET3* varied significantly between different survival statuses of AML patients (Figure 3E). We also reviewed the expression of m5C regulators based on treatment status and found that only *TRDMT1* is differentially expressed before and after treatment (Figure 3F).

### Genetic alterations associated with m5C regulators in AML

**Figure 4:**
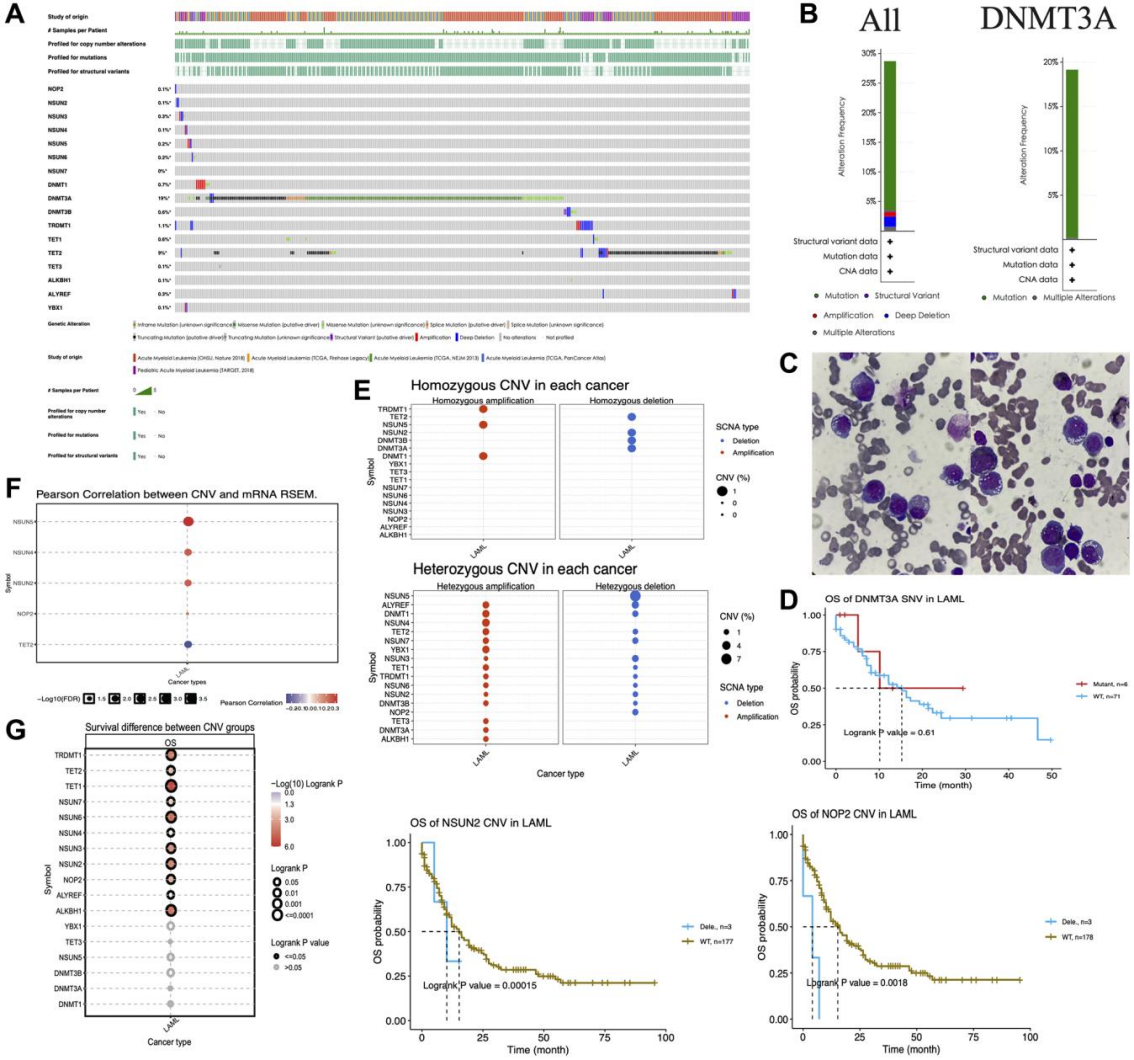
**Genetic alterations of m5C regulators in acute myeloid leukemia (AML).** (**A**) Mutation analysis of m5C regulators in AML patients. (**B**) Alteration frequency of m5C regulators and *DNMT3A*. (**C**) The peripheral blood smears of two AML patients. (**D**) Kaplan–Meier curves showing the overall survival of AML patients with *DNMT3A* mutations. (**E**) Homozygous and heterozygous CNV analysis of m5C regulators in AML patients. (**F**) Pearson correlation analysis between CNV and mRNA expression of m5C regulators. (**G**) Association between overall survival and deletions in m5C regulators in AML patients.

To elucidate the genetic alterations associated with m5C regulators, we integrated 4 datasets [24–26] from different studies using cBioPortal (http://www.cbioportal.org/) tools. We found that these regulators harbor a large number of mutations in AML patients (Figure 4A). Surprisingly, *DNMT3A* mutation rate was comparatively higher than other regulators and reached 19%, with missense mutations accounting for the majority of all mutations in this gene. *TET2* was the next with ~9% mutations, and it mainly harbored truncating mutations (Figure 4A). Figure 4B shows the alteration frequency of all m5C regulators together and *DNMT3A* alone. The peripheral blood smears of two AML patients showed morphologically aberrant red blood cells (Figure 4C). Genome sequencing of these two patients also identified mutations in *DNMT3A*.

Given this high mutation rate of *DNMT3A*, we performed Kaplan–Meier survival analysis to validate the effect of *DNMT3A* mutations. The findings suggested that *DNMT3A* mutation did not significantly affect the survival of AML patients (Figure 4D). The CNVs of m5C regulators were also analyzed in AML. We found that *TRDMT1*, *NSUN5* and *DNMT1* showed homozygous amplifications and *TET2, NSUN2, DNMT3B* and *DNMT3A* showed homozygous deletions in AML patients, while almost all m5C regulators showed heterozygous amplifications (except for *NSUN5* and *NOP2*) and heterozygous deletions (except for *NSUN4, YBX1, TET3, DNMT3A* and *ALKBH1*) (Figure 4E). We further explored the correlation between the CNV and mRNA expression, and the result suggested that the CNVs of *NSUN2, NSUN4, NUSN5* and *NOP2* were positively correlated with their mRNA expression levels, whereas *TET2* CNV was negatively correlated with *TET2* mRNA expression (Figure 4F). We further investigated whether deletions in m5C regulators affect the survival of AML patients. Our results revealed that deletions in *NSUN3, NOP2* and a few other regulators were significantly associated with worse prognosis of AML patients (Figure 4G).

### DNA methylation of m5C regulators in AML

**Figure 5:**
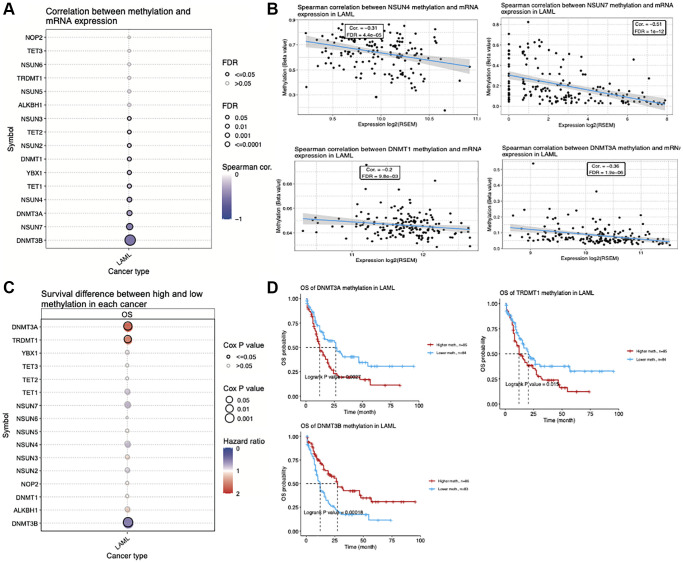
**Correlation between DNA methylation status and expression of m5C regulators in acute myeloid leukemia (AML).** (**A**) Correlation between the methylation status and mRNA expression of m5C regulators in AML. (**B**) Scatterplots showing correlation between methylation status and expression of four representative m5C regulators. (**C**) Association between overall survival of AML patients and methylation status of m5C regulators. (**D**) Kaplan–Meier curves showing the overall survival of AML patients with *DNMT3A*, *DNMT3B* and *TRDMT1* methylation.

DNA methylation is an epigenetic modification that is involved in the regulation of gene expression [27, 28]. We found that *TET2, NSUN2, DNMT1, YBX1, TET1, NSUN4, DNMT3A, NSUN7* and *DNMT3B* methylation levels were negatively correlated with their expression levels (Figure 5A). Figure 5B shows the Spearman’s correlation values between the methylation and expression levels of four representative genes (*NSUN4*: R = −0.31, FDR = 4.4e-05; *NSUN7*: R = −0.51, FDR = 1e-12; *DNMT3A*: R = −0.36, FDR = 1.9e-06; *DNMT1*: R = −0.20, FDR = 9.8e-03). Furthermore, the methylation levels of m5C regulators also affects the overall survival of AML patients (Figure 5C). Interestingly, we found that AML patients with high DNA methylation levels of *DNMT3A* and *TRDMT1* have a significantly shorter overall survival, while those with high DNA methylation levels of *DNMT3B* have a significantly longer overall survival (Figure 5C, 5D).

### Evaluation of clinical values of m5C regulated gene subgroups in AML using consensus clustering

**Figure 6:**
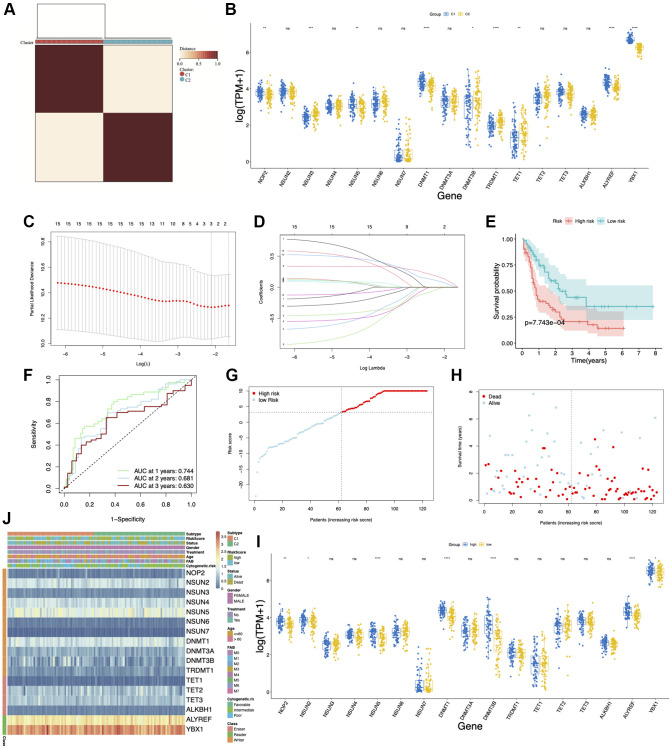
**Consensus clustering and prognostic model of m5C regulators in acute myeloid leukemia (AML).** (**A**) Consensus clustering matrix for k = 2. (**B**) Box plots showing the expression of m5C regulators in two clusters. (**C**, **D**) LASSO Cox regression algorithm was used for calculating the minimum criteria. (**E**) Kaplan–Meier curves showing the overall survival of AML patients with high- and low-risk scores. (**F**) ROC curve showing the AUC value of the model for different survival times. (**G**, **H**) Distribution of the risk score and survival status. (**I**) Box plots showing the expression of m5C regulators in high- and low-risk groups. (**J**) Heatmap and clinicopathological characteristics of AML molecular subtypes and high- and low-risk groups. ^*^p < 0.05, ^**^p < 0.01, ^***^p < 0.001.

To clarify whether the AML patients can be divided into different subgroups according to the expression of m5C regulators, we performed unsupervised clustering by ConsensusClusterPlus based on the 17 m5C regulators. Two m5C modification patterns (C1 and C2) were finally identified in AML patients (Figure 6A). *NOP2, NSUN3, NSUN5, DNMT1, DNMT3B, TRDMT1, TET1, ALYREF* and *YBX1* were differentially expressed in these two clusters (Figure 6B). The LASSO Cox regression algorithm was applied to these candidate genes in the AML cohort. Eventually, 11 genes were identified based on the criteria to construct the m5C modification signature prognostic model (Figure 6C, 6D). We further divided the AML patients into a high-risk score group and a low-risk score group based on the median risk scores. The K-M plots demonstrated that the low-risk score group survived longer than the high-risk score group (Figure 6E). The ROC curve analysis showed that the AUC of the prognostic model was 0.744, indicating that the model has a good predictive ability (Figure 6F). The risk score and clinical status from two risk groups are shown in Figure 6G, 6H. We also observed the expression of m5C regulators in the low-risk and high-risk group; *NOP2, NSUN2, NSUN5, DNMT1*, *DNMT3B, ALYREF* and *YBX1* were found to be highly expressed in the high-risk group (Figure 6I). In addition, there was a difference between the AML patients in the two cluster groups in terms of status, treatment, and cytogenetic risk (Figure 6J).

**Figure 7:**
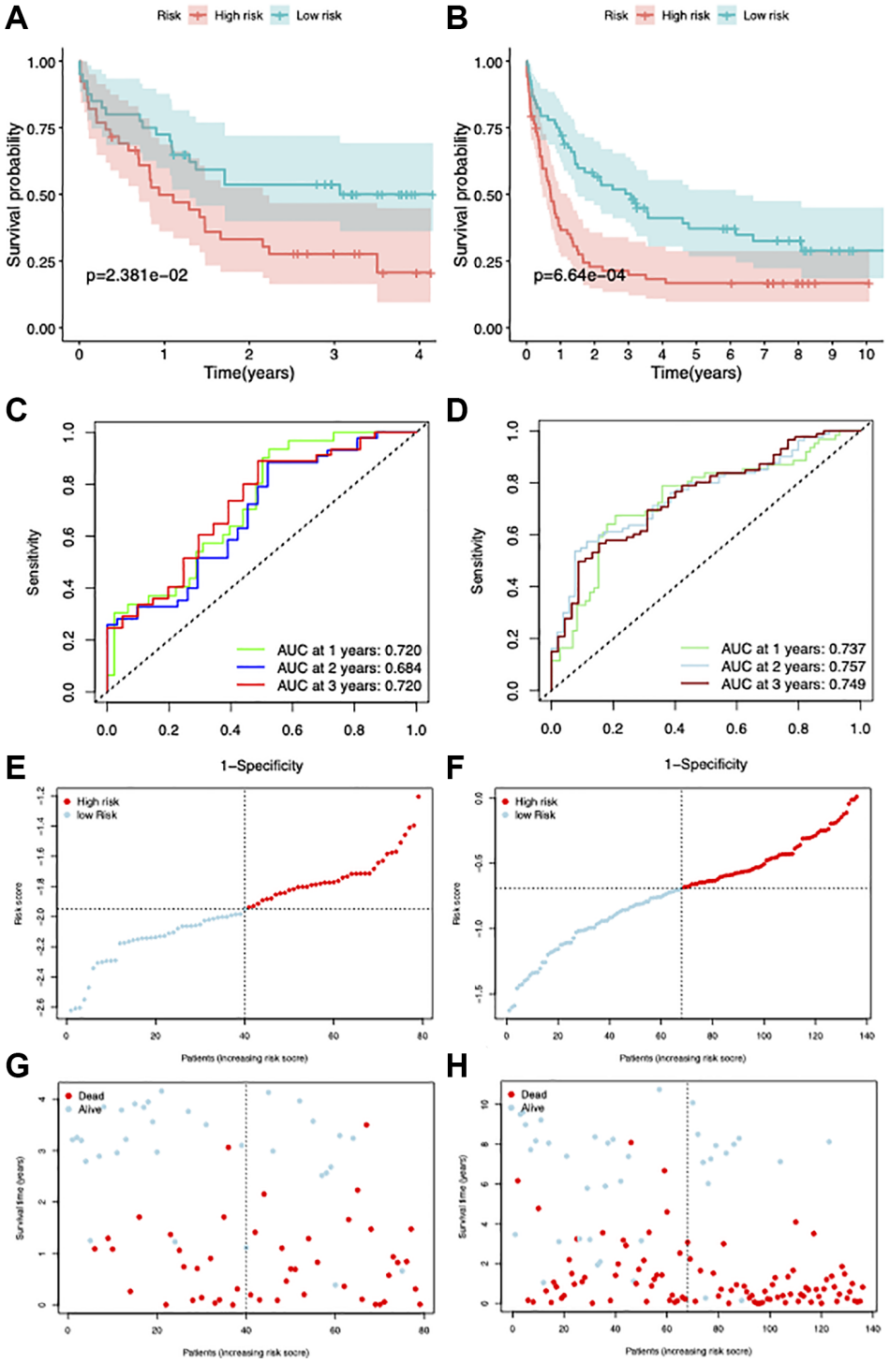
**Validation of the prognostic model.** (**A**, **B**) Survival curves of patients in high and low-risk groups in GSE12417 and GSE37642 datasets. (**C**, **D**) ROC curves showing the AUC value of the model for different survival times. (**E**–**H**) Distribution of the risk score and survival status.

To validate the stability of our model, GSE12417 [29] and GSE37642 [30] datasets were used. The survival statistics for the high- and low-risk groups showed that the proportion of surviving patients was comparatively higher in the low-risk group, which had a better prognosis (Figure 7A, 7B). The AUC values of this model were 0.720 and 0.757 for GSE12417 and GSE37642 datasets, respectively (Figure 7C, 7D). The risk score and clinical status of each case from the two risk groups are shown in Figure 7E–7H.

### Differential infiltration of immune cells in AML correlates with the cluster groups and risk model of m5C regulators

**Figure 8:**
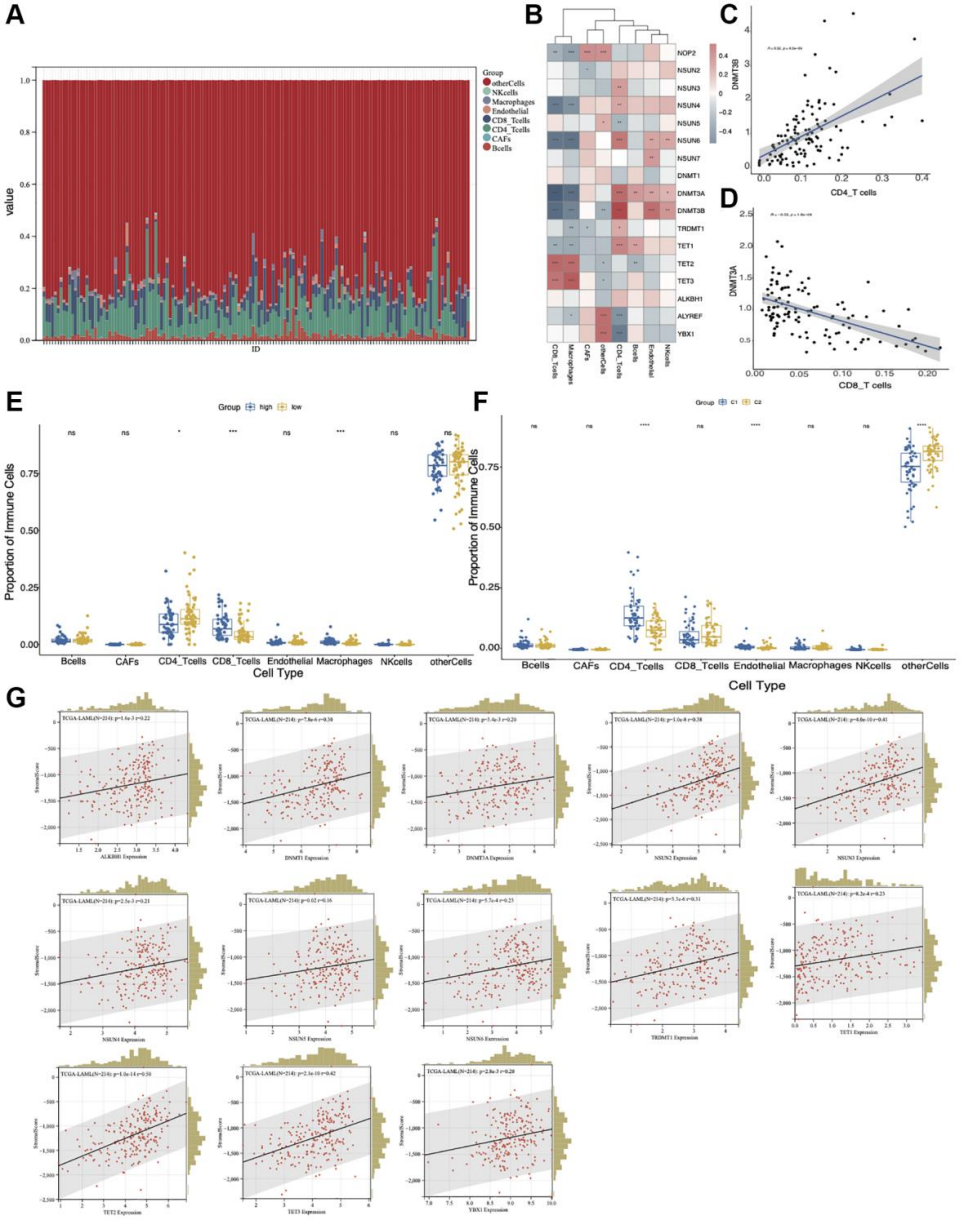
**Correlation of the immune cell infiltration with high- and low-risk groups and m5C regulators-based subtypes.** (**A**) The proportion of immune cells by EPIC algorithm. (**B**) The correlation between m5C regulators and different immune cell types. (**C**) *DNMT3B* is positively correlated with CD4 T-cells. (**D**) *DNMT3A* is negatively correlated with CD8 T-cells. (**E**) Differential infiltration of immune cell types between low- and high-risk groups. (**F**) Differential infiltration of immune cell types between C1 and C2 groups. (**G**) Correlation between m5C regulators and stromal score. ^*^p < 0.05, ^**^p < 0.01, ^***^p < 0.001.

To clarify the relationship between immune cell infiltration and m5C clusters or risk grouping, we used the EPIC algorithm to quantify the proportions of immune cells (Figure 8A). Further, we analyzed the correlation between m5C regulators and the infiltrated immune cell types (Figure 8B–8D). *NSUN4* and *NSUN6* were negatively correlated with CD8 T-cells and macrophages, and positively correlated with CD4 T-cells. *DNMT3A* and *DNMT3B* were negatively correlated with CD8 T-cells and macrophages, and positively correlated with CD4 T-cells, endothelial and NK cells. *TET1* and *TET2* were positively correlated with CD8 T-cells and macrophages. Next, we wanted to know whether there is a difference in the infiltration of various immune cells between the low- and high-risk groups. The result showed that CD4 T-cells were enriched in the low-risk group, while CD8 T-cells and macrophages were enriched in the high-risk group (Figure 8E). For different m5C subtypes, CD4 T-cells and endothelial cells were enriched in C1 cluster, while CD8 T-cells were enriched in C2 cluster (Figure 8F). To gain more insight into the relationship between immune infiltration and m5C regulators, we calculated stromal score of AML microenvironment by using ESTIMATE and evaluated the correlation between m5C regulators and stromal score. Our results suggested that *ALKBH1, DNMT1, DNMT3A, NSUN2, NSUN3, NSUN4, NSUN5, NSUN6, TRDMT1, TET1, TET2, TET3, YBX1* were positively correlated with the stromal score (Figure 8G). The above results suggest that infiltration of different immune cells does exist in high and low risk groups as well as in different m5C clusters. These differences in the proportion of immune cells may be an important factor affecting the prognosis of patients in different groups of AML patients.

### Relationship between m5C regulators and RNA based stemness score, drug sensitivity and miRNA expression

**Figure 9:**
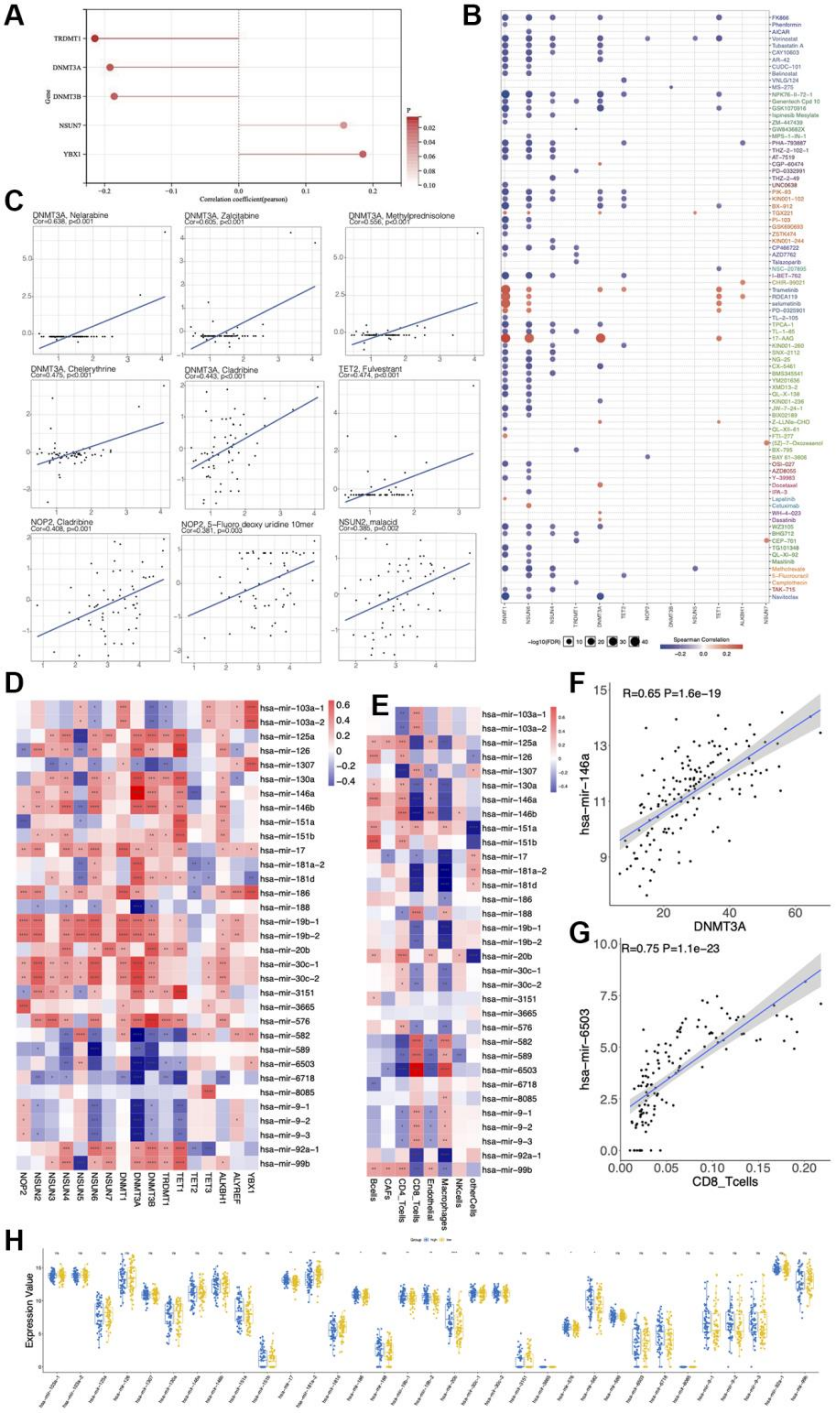
**Relationship between expression of m5C regulators and RNA based stemness score, miRNA expression and drug sensitivity.** (**A**) Correlation between m5C regulators and RNA based stemness score (RNAss). (**B**, **C**) Correlation between drug IC50 and m5C regulators. (**D**) Correlation between expressions of miRNAs and m5C regulators. (**E**) Correlation between immune cells and expressions of miRNAs. (**F**) has-mir-146a is positively correlated with *DNMT3A*. (**G**) has-mir-6503 is positively correlated with CD8 T cells. (**H**) Differential expression of miRNAs between low- and high-risk groups.

We also wondered whether those m5C regulators could affect other features of AML. Hence, we analyzed RNA Based Stemness Score (RNAss), miRNA expression and drug sensitivity. Our results suggested that *TRDMT1, DNMT3A* and *DNMT3B* were negatively correlated, while *NSUN7* and *YBX1* were positively correlated with the RNAss (Figure 9A). For the drug sensitivity analysis, we calculated the correlation between drug IC50 and gene expression. Our results revealed that m5C regulators were correlated with many drugs (Figure 9B). We found that *DNMT3A* was positively correlated with Nelarabine (R = 0.638, *p* < 0.001), Zalcitabine (R = 0.605, *p* < 0.001), Methylprednisolone (R = 0.556, *p* < 0.001), Chelerythine (R = 0.475, *p* < 0.001), Cladribine (R = 0.443, *p* < 0.001); *TET2* was positively correlated with Fulvestrant (R = 0.474, *p* < 0.01); *NOP2* was positively correlated with Cladribine (R = 0.408, *p* = 0.001), 5-Fluorodeoxyuridine 10mer (R = 0.381, *p* = 0.003); and *NSUN2* was positively correlated with malacid (R = 0.385, *p* = 0.002) (Figure 9C). Furthermore, we performed correlation analysis between all miRNAs and m5C regulators and found 33 miRNAs to be significantly correlated with m5C genes. We found *DNMT3A* to play a significant role in this gene-miRNA network, which positively regulated 13 miRNAs (*P* < 0.0001) and negatively regulated 7 miRNAs (*P* < 0.0001) (Figure 9D). Further CD8 T-cells played a key role in the immune cell-miRNA network, and positively regulated 4 miRNAs (*P* < 0.0001), while negatively regulated 5 miRNAs (*P* < 0.0001) (Figure 9E). As shown in the scatter plot, *DNMT3A* is positively correlated with hsa-mir-146a (R = 0.65, *P* = 1.6e-19) (Figure 9F) and CD8-T cells are positively correlated with hsa-mir-6503 (R = 0.75, *P* = 1.1e-23) (Figure 9G). The differential expression analysis of miRNAs between the low- and high-risk groups revealed hsa-mir-17, hsa-mir-186, hsa-mir-19b-1, hsa-mir-19b-2, hsa-mir-20b, hsa-mir-576 and hsa-mir-582 to be highly expressed, while hsa-mir-181a-2 to be lowly expressed in the high-risk group (Figure 9H).

### Pathways different between the high- and low-risk groups

**Figure 10:**
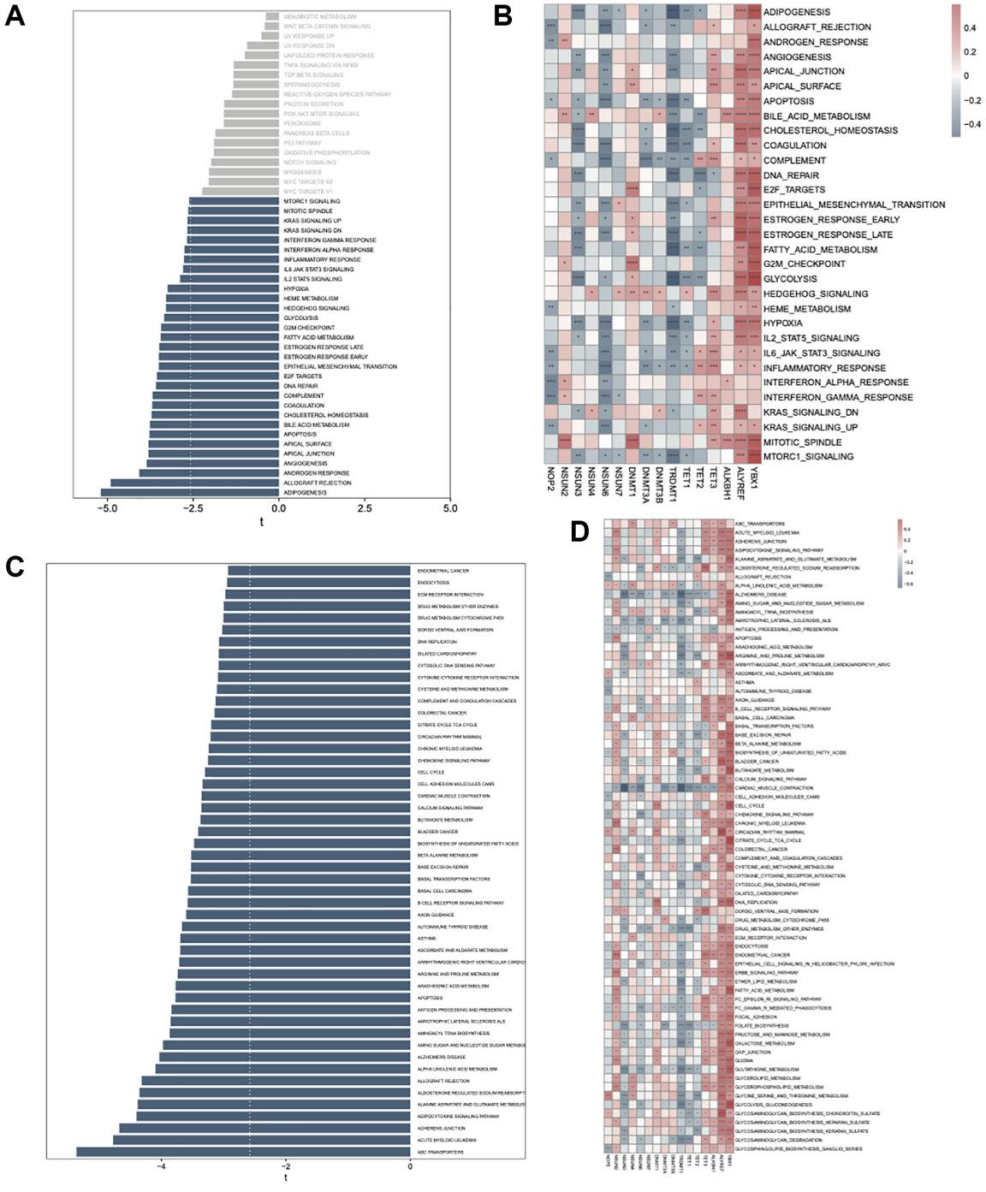
**GSVA analysis between high- and low-risk AML groups.** (**A**) Enrichment of hallmark pathways between high- and low-risk groups. (**B**) Correlation between the hallmark pathway scores enriched in high-risk group and m5C regulators. (**C**) Enrichment of KEGG pathways between high- and low-risk groups. (**D**) Correlation between the KEGG pathway scores enriched in high-risk group and m5C regulators.

The GSVA tool was used to analyze the differences in the KEGG and hallmark pathways between the high- and low- risk groups. Almost all the hallmark and KEGG pathways were found to be enriched in the high-risk group (Figure 10A, 10C). Further, we performed correlation analyses between the gene expression values and pathway scores. For the hallmark pathways enriched in the high-risk group, we found *NOP2*, *NSUN3*, *NSUN6*, *TRDMT1*, and *TET1* to be significantly negatively correlated with most pathways, while *TET3*, *ALYREF*, and *YBX1* to be significantly positively correlated (Figure 10B). For KEGG pathways enriched in the high-risk group, we found *TRDMT1* and *TET1* to be significantly negatively correlated, while *ALYREF*, and *YBX1* to be significantly positively correlated with most of the pathways (Figure 10D).

### Expression levels of m5C regulators in specific cell types

**Figure 11:**
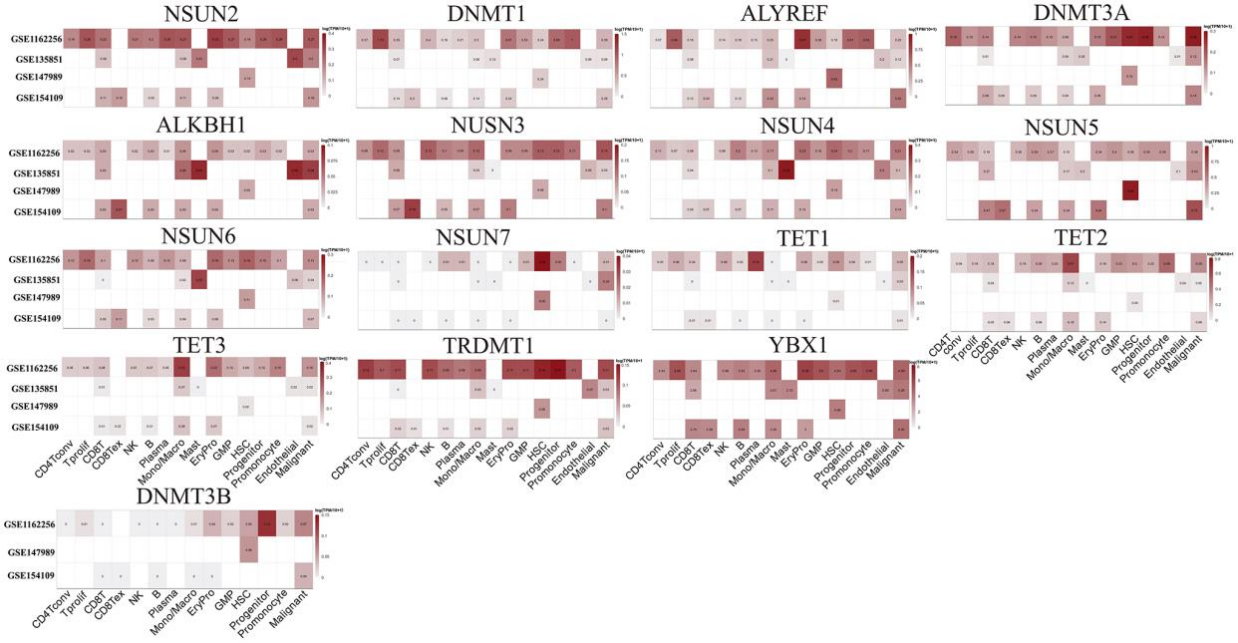
Expression levels of m5C regulators in specific cell types based on single cell expression data.

We downloaded the cell type specific expression data including scRNA-seq data from GSE116256 [31], GSE135851 [32], GSE147989 [33], and GSE154109 [34] datasets to illustrate the expression of m5C regulators in specific cells (Figure 11). Based on these four datasets, we identified the expression of the genes in the following cells: conventional CD4 T cells (referred as CD4Tconv), proliferating T cells (Tprolif), CD8 T cells (CD8T), exhausted CD8 T Cells (CD8Tex), natural killer cells (NK), B cells (B), plasma cells (Plasma), monocytes or macrophages (Mono/Macro), mast cells (Mast), erythroid progenitor cells (EryPro), granulocyte-macrophage progenitor cells (GMP), hematopoietic stem cells (HSC), progenitor cells (Progenitor), promonocytes (Promonocyte), endothelial cells (Endothelial) and malignant cells (Malignant). At single-cell level, the expressions of *ALYREF*, *DNMT1*, *NSUN5*, and *YBX1* were higher than other genes. Furthermore, *ALYREF* was found to be enriched in EryPro, *DNMT1* was enriched in Tprolif and Promonocyte, *NSUN5* was enriched in HSC, while *YBX1* was enriched in almost all the cells (Figure 11).

### RT-qPCR validation of the selected m5C regulators

**Figure 12:**
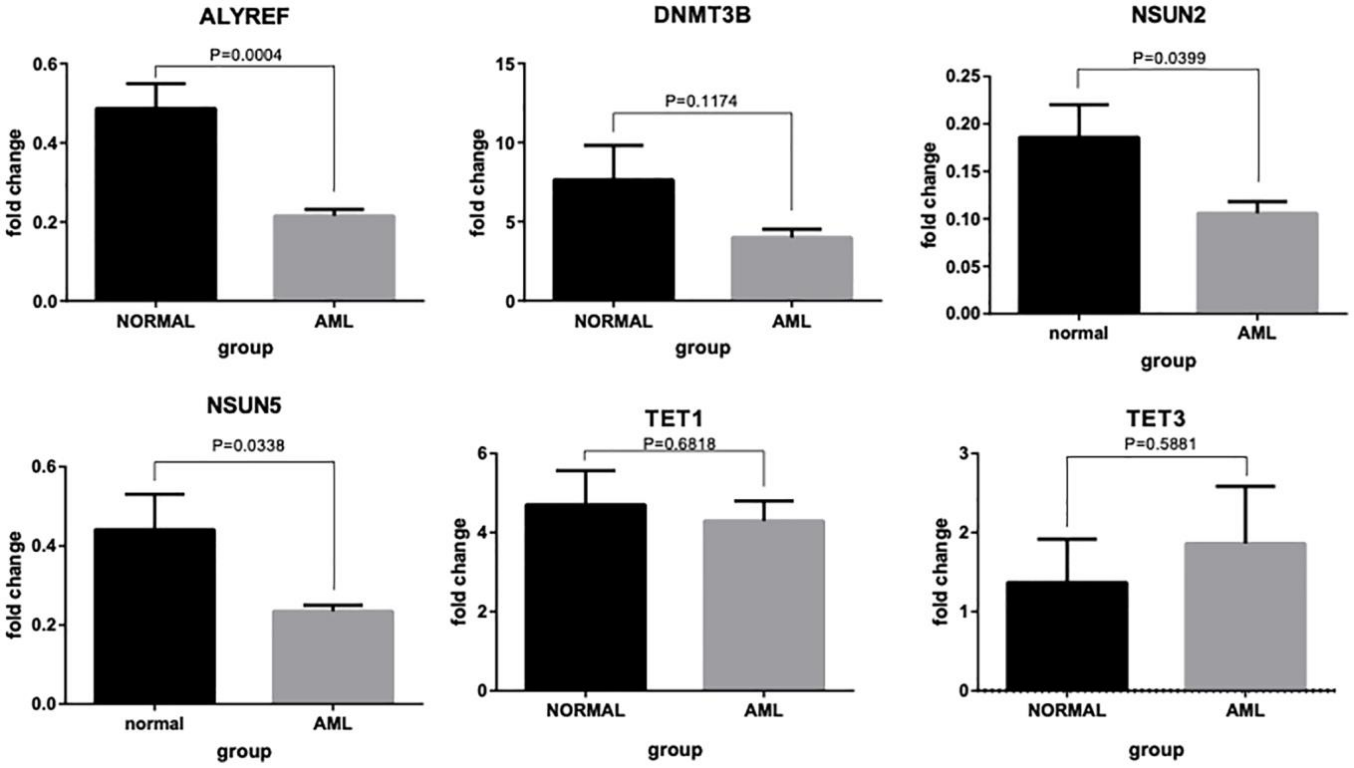
**RT-qPCR validation of six m5C regulator genes.** Bar plots showing differential expressions of *ALYREF*, *DNMT3B, NSUN2, NUSN5, TET1* and *TET3* between AML patients and normal controls.

To verify the reliability of our analysis and conclusions, six molecules (*ALYREF*, *DNMT3B, NSUN2, NUSN5, TET1* and *TET3*) were selected further to confirm their expression levels in AML versus normal tissues using qRT-PCR (Figure 12). The results showed that the expression levels of *ALYREF, NSUN2,* and *NUSN5* were significantly higher in normal controls than in AML patients. This is in agreement with the results of our other analyses. Although we did not observe any significant differences in the expression levels of *DNMT3B, TET1* and *TET3* between the normal and AML samples, the trends in their expression levels were quite consistent with our other analysis results. Thus, the partial disagreement in the qRT-PCR results could be attributed to the heterogeneity of AML patients.

## DISCUSSION

Recently, with the development of high-throughput sequencing technology, RNA modification has gradually become an important research area [22, 35]. (m6A) is the most abundant internal modification of mRNA and has been studied in depth [36–38]. Zhang et al. found that m6A reader *IGF2BP3*, which interacts with and enhances the *RCC2* mRNA stability, is essential for AML cell survival [39]. Yankova et al. suggested that inhibition of *METTL3* could be a potential target for the treatment of AML [40]. m5C modifications have also been found to play regulatory roles in a variety of biological processes, particularly in tumor progression. Compared to m6A modifications, m5C modifications have been less studied in AML. Cheng et al. reported the mechanism of m5C methylation in AML drug resistance [41]. Liu et al., [42] collected and analyzed multicenter AML data to suggest that not only AML diversity but also generation of complex tumor microenvironments are impacted by m5C modifications. Mutations in the well-known DNA methyltransferase gene *DNMT3A* are highly recurrent in patients with *de novo* AML and are known to be independently associated with a poor outcome [43]. More recently, 5-hydroxymethyl cytosine (5-hmC) has garnered lot of attention as a regulatory epigenetic modification with diagnostic and prognostic significance for several cancers [44]. TET family of proteins can oxidize m5C to 5hmC, which is known to play important roles in the pathogenesis of various tumors including AML [45, 46]. Both *TET2* and *IDH1/2* mutations can impair the production of 5hmC, thus decreasing 5hmC levels. Mutant IDH enzymes increase the production of oncometabolite (R)-2-hydroxyglutarate that competitively inhibits dioxygenase enzymes that are required for m5C to 5-hmC modification and histone tail methylation [47]. Mutations in both these genes have been observed in AML [48]. Overall, the role of m5C modifications in AML remains ambiguous and limited. The present study focuses on investigating the multifaceted characteristics of m5C regulators in AML with the aim of contributing new evidence to the field of AML research.

Network analysis of various molecular and regulatory factors [49, 50] is a strong and widely used approach for exploring the underlying mechanisms of any disease. It has not only been used in AML research [51–53] but also for several other diseases and physiological conditions [54–56]. In the current study, we constructed protein-protein interaction network and revealed the close relationships among the m5C regulators. Additionally, studying the interactions by combining various gene-regulatory molecules including TFs, non-coding RNAs, and m5C regulators could expand our understanding of the regulation of these factors. Furthermore, construction of the independent networks for high- and low-risk AML groups may help us in gaining better insights into the differential role of these m5C regulators as risk factors.

The analysis of molecular subtypes based on some features has been widely used in tumor research. The molecular subtypes can cluster cancer patients into different groups that have different characteristics (e.g., different sensitivity to drugs). Based on such subtypes, cancer patients can be diagnosed and treated more precisely. Jayavelu et al. classified AML into five subtypes based on proteogenomic features; they identified a “Mito-AML” group, which is characterized by high expression of mitochondrial proteins and poor prognosis [57]. Mou et al. identified molecular subtype- specific mRNA expression patterns and studied the characteristics of different subtypes in depth [58]. Mer et al. reported a subtype with *NPM1* mutation, which was based on stemness, and suggested applied kinase inhibitors for the treatment of this subtype [59]. However, whether m5C regulators can classify AML patients into different subtypes has not been explored till date. In the present study, AML patients were classified into C1 and C2 subgroups using m5C regulators, and significant differential gene expression and prognosis between the subgroups could be observed. Therefore, it is of great interest to further explore the differences between these two subtypes.

In recent years, with the advancement of immunological research, immunotherapy has been applied to the treatment of AML. These patients have a comparable number of T cells in bone marrow (BM); the percentage of CD3+ and CD8+ T-cells in BM could predict response to the treatments [60]. Williams et al. showed that NK cell-based therapy has a promise to improve the drug response and survival of AML patients [61]. Myeloid-derived suppressor cells (MDSCs) have also been found to dramatically increase in AML patients [62, 63]. MDSCs are proliferated by inducing AML cells through the extracellular vesicle (EVs), which contain several proliferation factors (e.g., *MUC1*) [64]. In the present study, we were also curious about the changes in the proportion of immune cells in different AML patient subgroups. Therefore, we used the EPIC algorithm for deconvolution to infer immune cell infiltration. We found differences in the proportion of immune cells across molecular subtypes as well as high- or low- risk groups, suggesting that further exploration of the mechanisms of immune cell changes in m5C subtype may provide novel insights into the treatment of AML.

Our drug sensitivity analysis identified several drugs to be significantly correlated with the m5C regulators. For example, *DNMT3A* correlated with Nelarabine, which is an FDA approved drug and is used for the treatment of hematologic malignancies [65]. Zalcitabine, IC50 value of which was positively correlated with *DNMT3A* expression has been FDA approved for the treatment of HIV/AIDS [66], while Methylprednisolone is an FDA-approved medication for the management and treatment of allergic conditions and acts as an anti-inflammatory and immunosuppressive agent [67]. Fulvestrant, an antiestrogenic medication that is used for the treatment of receptor-positive metastatic breast cancer was found to be positively correlated with *TET2* [68]. Cladribine, an FDA approved drug correlated with both *DNMT3A* and *NOP2*. It is currently used for the treatment of hematologic malignancies and a few other diseases [69, 70]. Malacid (Pyrimethamine), which significantly correlated with *NSUN2* expression is mainly used for fungal and parasitic infection [71]. The strong positive correlation of these drugs with m5C regulators indicates that many of these that are currently used for other diseases and conditions can be explored as a therapeutic option for AML. Furthermore, the analysis of antagonistic and coordinated effects between drugs will provide references for precise medical treatment of LAML [72].

Certainly, although several meaningful results were obtained in the present study, there were a few limitations. Some of the important molecules we found (e.g., *DNMT3A*) require further validation using other datasets as well as experiments. Additional external validations based on other cohorts are needed to evaluate whether the m5C molecular subtype and risk-score still perform well in AML patients. In addition, this study indirectly revealed the role of m5C modification in AML. In the future, we will use m5C MeRIP-seq technology to globally map m5C modifications in AML and explore the changes in modification levels in different m5C subtypes as well as in high- and low-risk groups.

## MATERIALS AND METHODS

### Data source

In this study, we downloaded the standardized data from the UCSC (https://xenabrowser.net/) database. The mRNA expression profiles, mutation annotation data, CNV data and clinical metadata were also downloaded from the UCSC database. The samples with incomplete clinical data were removed.

### Gene expression analysis of m5C regulators and survival analysis

Based on existing literature, we collected 17 m5C regulators. We employed the “inSilicoMerging” package to merge GTEx and TCGA database based on the matrixes of all gene expressions [73], and removed the batch effects by empirical bayes algorithm [74], and used the “*t*-test” algorithm to compare the differential expression of those m5C regulators. Pearson correlation analysis was used to measure the correlation among the m5C regulators. The univariate Cox regression was implemented to analyze the relationship of each m5C regulators’ expression with overall survival.

### Analysis of the CNV data and mutation annotation data

The CNVs of m5C regulators, including homozygous and heterozygous amplifications and deletions, were visualized using the bubble plot. The mutation annotation data of AML was used for further analysis with the R package “maftools” [75].

### Identification of m5C molecular clustering

We used the 17 m5C regulators for identifying m5C patterns in AML patients. Consensus clustering was performed by using R package “ConsensusClusterPlus” [76] to identify different clusters based on the differential expression of the m5C regulators. The number of classifications was chosen according to the area under the CDF curve and the k-value. The classification step was repeated 1000 times to increase the reliability.

### m5C regulators risk model construction

We applied the “glmnet” (https://glmnet.stanford.edu/articles/glmnet.html) [77] and ‘survival’ (https://github.com/therneau/survival) [78] packages for the least absolute shrinkage and selection operator (LASSO) regression to further screen the candidate m5C regulators, and finally a new m5C regulator signature was established. Based on the results of LASSO regression, we developed a risk score formula, which was calculated as follows:

RiskScore = −0.3202 × *NSUN3* − 0.2526 × *NSUN4* + 0.0372 × *NSUN5* − 0.3514 × *NSUN6* + 0.3160 × *NSUN7* + 0.3244 × *DNMT1* −0.3648 × *DNMT3A* + 0.3476 × *DNMT3B* −0.0334 × *TET2* − 0.0897 × *TET3* + 0.2137 × *ALKBH1*.

In addition, we clustered AML patients into high-risk and low-risk groups based on the median risk score. Then, GSE12417 and GSE37642 datasets were used to validate the established risk model.

### Assessment of infiltrated immune cells and correlation analysis

To clarify the immune infiltration differences between different m5C clusters and different risk groups, the “EPIC” algorithm [79] was used to evaluate the score of 8 immune cells. We further applied Wilcox test to compare the differences in immune cell score between different m5C clusters, and high and low risk score groups. Additionally, we applied the ESTIMATE algorithm to calculate the stromal infiltration and then correlation was calculated between m5C regulators and stromal score.

### Analysis of miRNA co-expression and drug sensitivity of m5C regulators in AML

We obtained miRNA expression data of AML patients using the TCGA database. Candidate miRNA-m5C regulator pairs were obtained from the ENCORI database [80]. Pearson correlation analysis was performed to calculate the correlation between miRNA expression and that of m5C regulators. The drug sensitivity data were acquired from The Genomics of Drug Sensitivity in Cancer (GDSC) database (https://www.cancerrxgene.org/). We downloaded the IC50 values of each drug using the R package “pRRophetic” [24]. Then, correlation analysis was performed between the sensitivity and the expression of m5C regulators.

### GSVA and correlation analysis

GSVA was used to analyze the KEGG and hallmark pathway scores between high-risk and low-risk subtypes [81]. We calculated the correlations between m5C regulators and KEGG or hallmark pathway scores.

### Real-time quantitative reverse transcription polymerase chain reaction (RT-qPCR)

Peripheral blood mononuclear cells were isolated from AML patients and the patient’s healthy family members (*n* = 4) from the Affiliated Hospital of Nantong University for *in vivo* testing. RNA was extracted using the MiniBEST Universal RNA Extraction Kit (Takara Bio, USA), followed by reverse transcription using a HiScript III 1st Strand cDNA Synthesis Kit (gDNA digester plus) and RT-qPCR. The primers used for the amplification are the following:

5′-CGTGGAGACAGGTGGGAAAC-3′ (forward) 5′-GTTCCTAAGCTGCGACCAGA-3′ (reverse) for *ALYREF*, 5′-ACAGAAAAGGAATGTGTGAAGGA-3′ (forward) 5′-TGGAATAGGGGACCTCGTGT-3′ (reverse) for *DNMT3B*, 5′-GACATAGCCTGTCGCTTGGA-3′ (forward) 5′-ATCCGCATAAGACGATGGGAC-3′ (reverse) for *NSUN2*, 5′-CGTGGAGACAGGTGGGAAAC-3′ (forward) 5′-TCCAGCAACTTCCAGAACGTGA-3′ (reverse) for *NSUN5*, 5′-TCAAATCTGGGGCCATCGAG-3′ (forward) 5′-TCATCATCGCAGCCCTCTTC-3′ (reverse) for *TET1*, 5′-CCACCAGCCTCTTTTGGGAA-3′ (forward) 5′-GCTCTGCTACTTCTTTCCTTGC-3′ (reverse) for *TET3*.

The result of the experiment was represented by relative quantitative analysis of 2^−ΔΔCT^. *ABL1* was used as control gene for normalizing the gene expression.

### Data availability statement

Data of mRNA expression profiles, mutation annotation data, CNV data and clinical metadata were from TCGA-LAML in UCSC (https://xenabrowser.net/). RNA-seq data have been deposited in the Gene Expression Omnibus (GEO) with accession numbers GSE12417 and GSE37642. scRNA-seq data have been deposited in GEO with accession numbers GSE116256, GSE135851, GSE147989, and GSE154109.

### Code availability statement

The full code used during the current study is available at https://github.com/NTUWangLab/M5C_LAML.
